# Advances in surfaces and osseointegration in implantology. Biomimetic surfaces

**DOI:** 10.4317/medoral.20353

**Published:** 2015-02-07

**Authors:** Matteo Albertini, Marc Fernandez-Yague, Pedro Lázaro, Mariano Herrero-Climent, Jose-Vicente Rios-Santos, Pedro Bullon, Francisco-Javier Gil

**Affiliations:** 1 Padrós Dental Institut, Marti i Julià, 6-8 bajos, 08034 Barcelona, Spain; 2 ETSEIB, Universitat Politècnica de Catalunya, Avda. Diagonal 647, 08028 Barcelona, Spain; 3 Faculty of Dentistry, Universidad de Sevilla, C/. Avicena s/n, 41004 Seville, Spain; 4 Department of Materials Science and Metallurgical Engineering (ETSEIB), Universitat Politècnica de Catalunya, Avda. Diagonal 647, 08028 Barcelona, Spain

## Abstract

The present work is a revision of the processes occurring in osseointegration of titanium dental implants according to different types of surfaces -namely, polished surfaces, rough surfaces obtained from subtraction methods, as well as the new hydroxyapatite biomimetic surfaces obtained from thermochemical processes. Hydroxyapatite’s high plasma-projection temperatures have proven to prevent the formation of crystalline apatite on the titanium dental implant, but lead to the formation of amorphous calcium phosphate (i.e., with no crystal structure) instead. This layer produce some osseointegration yet the calcium phosphate layer will eventually dissolve and leave a gap between the bone and the dental implant, thus leading to osseointegration failure due to bacterial colonization. A new surface -recently obtained by thermochemical processes- produces, by crystallization, a layer of apatite with the same mineral content as human bone that is chemically bonded to the titanium surface. Osseointegration speed was tested by means of minipigs, showing bone formation after 3 to 4 weeks, with the security that a dental implant can be loaded. This surface can be an excellent candidate for immediate or early loading procedures.

** Key words:**Dental implants, implants surfaces, osseointegration, biomimetics surfaces.

## Introduction

Dental implants represent a valid therapeutic option for the replacement of missing teeth (1). Developments in implantology have allowed to extend the reach of dental treatments through implant placement, since the latter provide long-term stable support for a dental prosthesis subjected to chewing load (2).

The biological principles followed for implant placement have already been described by some authors and can be summarized in the concept of osseointegration, which is defined as the direct and structural connection between living and structured bone, and the surface of an implant subjected to a functional load (3).

The earliest studies on this phenomenon were developed by Branemark in the 1950s, 1960s and 1970s, as well as by Schröeder (4), who proved that the alveolar bone is capable of forming a direct connection with a bolt-shaped alloplastic material such as titanium after being placed on a surgically-created bed.

Since implantology’s earliest stages, the growing interest of clinicians in this type of treatment has impelled research from the knowledge of the biological principles to the basis of osseointegration. A concept emerging from the studies by Johansson and Albrektsson is that osseointegration is a time-related phenomenon. Rigidity in bone-implant interface increases with time until reaching a high level 3 months after implant placement, and can increase progressively until 12 months after placement (5).

The time necessary for implant osseointegration is variable, as it depends on a series of factors that in turn depend, in one hand, on the bone and in other hand, on implant features. According to Branemark’s (3) protocol, waiting time for implant loading traditionally ranged from 3 to 6 months, depending on the implant’s maxillary or jawbone position. The implants used back then were made of commercially-pure titanium obtained by bar mechanization, and their surface topography resulted from their drilling process and their subsequent electrolytic polishing, thus being known as smooth or mechanized surface.

The implant’s surface features have been proven to influence the cicatrization of the bone surrounding it (6), and the use of rough surfaces as proven -by Beagle’s histological studies on dogs- showed that osseointegration can be achieved in a 6-week period under normal conditions with rough surfaces obtained through subtraction methods (7).

The morphology of these surfaces is involved in a series of biological events occurring after implant placement, which range from protein adhesion to peri-implant bone remodeling. These phenomena are favored by a particular surface roughness, thus allowing quicker osseointegration, which -from a clinical viewpoint- grants space for prosthesis placement within shorter time-periods.

Immediate or early implant loading is a procedure that has been back in use with good medium-to-long-term results in the last years (8). This is partly due to the use of implants with a more osteophilic surface, which allows maintaining implant stability more effectively throughout the first weeks of osseointegration. Reduction in implant primary stability due to initial bone resorption is counterbalanced by quicker bone neoformation, which lead to increased secondary stability and more predictable osseointegration.

Implant surface treatment is aimed at providing it with some particular features involving an excellent biological response in the surrounding tissue. There are several methods for dental implant surface treatment such as mechanizing, electropolishing, plasma spraying, coating, acid etching, surface oxidation, ionization, phosphate deposit techniques in some apathetic cases, or any combination of them (9).

Implant surfaces can mainly be classified into three main categories according to their biological response: Bioinert, osseoconductive and bioactive surfaces. The first are those around which bone cicatrization occurs from the bone to implant surface (slow cicatrization). The second are characterized by the fact that their surface morphology allows them to produce bone neoformation on implant surface (i.e., the bone starts forming from the surface to the periphery). These can present different roughness degrees and/or topographies that favor interaction with the proteins that promote migration of osteoblast precursor cells depending on their surface processing received.

Bioactive surfaces are those around which rapid bone neoformation occurs from implant surface, and are characterized by their surface showing -apart from different roughness degrees- some bioactive molecules or growth factors that induce bone formation according to different action mechanisms.

A bioactive implant surface -recently developed and based on the experimental studies by Pattanayak *et al*. (10)- can imitate osteoblast’s formation of the bone mineral part in its early stages. This is possible thanks to the development of a new thermochemical treatment of titanium that creates a calcium phosphate layer once in contact with biological fluids and prior to the arrival of osteoblastic cells. The use of implants with this type of biomimetic surface would allow quicker and more reliable osseointegration for cases of immediate or early implant loading.

The present paper is aimed at updating osseointegration mechanisms through the description of tissue response to different implant surfaces, as well as introducing the concept of the new biomimetic surface obtained by means of thermochemical methods.

## Implant osseointegration. Present-day concepts

-Present-day concepts

The bone is a mineralized connective tissue particularly structured to bear mechanical loads. Direct and structural connection between the living bone and the surface of an implant subjected to functional load was defined as osseointegration by Branemark (3). This phenomenon has been described and researched since the 1950s and still generates interest in modern implantology.

The most widely-re searched alloplastic material for dental implant manufacture is pure titanium and its alloy Ti6Al4V, always bolt-shaped. Titanium presents good biocompatibility, resistance to corrosion, and excellent mechanical properties. Implant surface osseointegration is what allows the implant to be subjected to chewing loads, which are transmitted to the bone.

Osseointegration as described by Branemark (3) is a clinical concept referred more to the stability of the implant subjected to chewing loading and in close contact with the bone rather than to the true microscopic joint of bone tissue and implant surface. This joint is the consequence of the biological events that lead to the interaction of bone cells with implant surface after surgical trauma.

The bone reacts to implant placement with a cicatrization process that is very similar to intramembranous ossification produced after bone fracture, except that the neoformed bone is in contact with the surface of an alloplastic material -the implant.

We can mainly recognize different biological events during implant-surrounding bone cicatrization -protein resorption, clot formation, granulation tissue formation, provisional matrix formation, interface formation, apposition and bone remodeling.

-Protein adsorption

In a first moment after dental implant placement, the latter is blood soaked and the present proteins present will subsequently be absorbed by its surface. The degree of wetting of the implant surface plays a relevant role in blood protein adsorption, since it has been proven that both excessive hydrophilia -unlike generally thought- or hydrophobia, hinders protein adsorption (11). Indeed, both highly hydrophilic and extremely hydrophobic surfaces allow no formation of a liquid drop with enough volume for proteins to be absorbed by the implant surface. Once blood can ideally soak implant surface, proteins (cytokines) can be absorbed and remain on the surface to work as a signal for the migration of osteoblastic cell lines, which will form the new bone around the implant and allow implant osseointegration.

Subsequently, neutrophils and macrophages question the implant and -according to the formation, orientation and type of absorbed proteins (12)- macrophages interact with implant surface and segregate a particular type and number of cytokines (biological molecular messengers) that can either gather the osteoblastic cell line in charge of bone formation in direct contact with surface implant, or the fibroblast cell line that encapsulates biomaterial in fibrous connective tissues and results in osseointegration failure.

Protein adsorption occurs practically instantaneously, thus inhibiting direct cell-biomaterial contact. Indeed, after exposing the surface to contact with blood, adsorption time is around 5 seconds (13).

Implant surface’s nature of one only layer of absorbed proteins constitutes the key factor of cell response, since cells have been proven to depend on specific proteins to adhere themselves (14). Particularly, osteoblasts demand specific interactions to adhere, proliferate and differentiate, and these interactions are defined by the number and type of proteins adsorbed in implant surface. Implant surface’s chemical and topographic nature will determine protein adsorption and conformation in (its) surface (15).

-Types of proteins

For osteoblasts to be able to onset bone formation around the implant, they must previously adhere themselves to implant surface. In vitro studies observed that these cells’ adhesion depends on some specific proteins absorbed in implant surfaces such as fibronectin, osteopontin and vitronectin. The last protein, proved in in-vitro and in-vivo studies, as the one that usually predominates in cell adhesion processes, followed by fibronectin (16) ( Table 1). However, the latter usually acquires more and more relevance once cells onset their differentiation process (17).

**Table 1:**
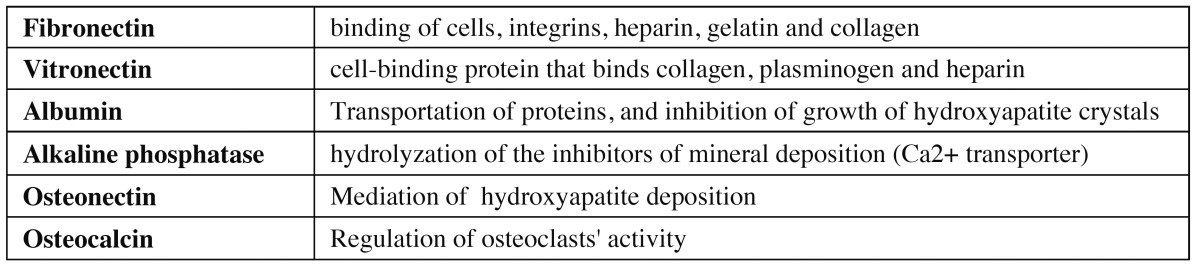
Proteins and their functions.

Implant surfaces play a determining role in the first stages of cell adhesion, since it is their topographic and physicochemical features that are capable of inhibiting the adsorption of the proteins that facilitate the migration of the undesired cells that provoke implant fibrointegration. TGF-α is an example of this, since it is a protein that favors fibroblastic cell line adhesion. For instance, fibroblasts can trigger migration to the implant of undesired cells (18).

Pegueroles *et al*. (11,15) proved in an in-vitro study that surface treatment of titanium dental implants with a specific size (A6) of alumina sand improves fibronectin adsorption relative to smooth titanium surfaces.

-Cell-protein interaction

Cells are capable of interacting with proteins by means of cell receptors known as integrins. However, integrin-protein interactions are completed through recognition of a particular amino acid sequence within a protein by the integrin. This is the case of the RGD amino acid (Arg-Gly-Asp) sequence present in adhesive proteins such as fibronectin. Integrin-protein interaction determines the regulation of multiple cell functions such as adhesion. Ramaglia *et al*. proved that osteoblasts change integrin's expression according to implant surface's chemical composition and roughness degree, where alumina sanded and acid etched surfaces showed greater expression relative to smooth surfaces (19).

After this first protein adsorption stage, the arrival of polymorphonuclear neutrophils and macrophages to the implant surface occurs. These generate a cascade of intercellular signaling that shall derivate in implant acceptance or refusal according to the recruited cells.

## Blood clot formation

Some minutes after implant insertion into the bed, a blood clot forms between the implant surface and the bone walls of the created bed. This mainly contains red blood cells, platelets and macrophages in a fibrin scaffold. During the first days a series of cytokines or growth factors (PDGF, TNFα, TGFα, TGFβ, FGF, EGF) are released to stimulate healing of the surgical wound gathering different cell lines. Two to three days after implant placement, leukocytes and macrophages complete ‘cleaning’ tasks through the phagocytosis process and the blood clot is simultaneously deconstructed through fibrinolysis to leave space for new blood vessels.

-Granulation tissue formation

Four days after placement, blood vessel growth produces a granulation tissue that occupies the space between the implant and the bone. This tissue is characterized by the presence of non-differenced mesenchymal cells around vessel structures in a fibrin scaffold. Surgical bed preparation -due to tissue trauma itself, which releases specific cytokines such as BMP2 and BMP4- induces the differentiation of non-differentiated mesenchymal cells in the bone marrow and peri-vascular (pericytes) firstly in pre-osteoblasts and subsequently in mature osteoblasts.

-Provisional matrix formation

Osteoblastic cells physically move in the space between the bone and the implant, and their migration is guided by the fibrin scaffold. In osseoconductive surfaces such as, for instance, those obtained by blasting and acid etching, cells adhere themselves to the proteins absorbed in implant surface and start forming a provisional bone matrix (20).

Osteoblasts are incapable of producing matrix and move simultaneously, so they stop migrating along the fibrin scaffold once they have started to produce the bone matrix. If the fibrin scaffold is removed from the implant surface during migration, osteoblasts will not reach it directly and no bone formation will therefore take place from the implant surface (20). However, fibrin adhesion to implant depends on the implant’s type of surface. On those of smooth or mechanized titanium, fibrin is removed during osteoblast migration, while in rough surfaces fibrin’s adhesion force is higher and cells can migrate to reach implant surface.

Thus, two main types of osseointegration can be distinguished: contact osseogenesis as described by Osborn *et al*. (21), in which progressive contact between the bone neoformed from the periphery to the implant bed; and the bone neoformation described by Davies *et al*. (22), where osteoblasts that can migrate to the implant surface through the fibrin scaffold (.) form new bone from the implant back to the bed walls.

-Bone apposition

Bone neoformation starts in early cicatrization stages, and after 7 days a provisional matrix rich in collagen fibers, vascular structures, osteoblasts and some neoformed bone area (bone apposition) begin to form (23). Some growth factors such as BMP 2 and 4 take part by stimulating the later migration of non-differentiated mesenchymal cells and by differentiating osteoblasts (BMP 7). After 14 days the implant-bone gap is occupied by neoformed or woven bone, which is rich in collagen fibers, vascular structures and osteoblasts, which form a reticular structure. In this stage osteoblasts produce the interface bone and can be found, in parallel to the surface, in the osseoconductive surfaces in contact with the implant. Bone neoformation on implant surface in early stages seems more characteristic of rough surfaces than of mechanized titanium (23). At the centre of the neoformed bone tissue some osteocytes can be observed while osteoclasts appear on bed bone surface, thus indicating necrotic bone resorption.

During the apposition process, bone structure progressively transforms from reticular to lamellar. Reticular bone is fragile and poor in calcium phosphate crystals, and transforms firstly into bone rich in parallel fibers and then into lamellar bone, which is mineralized tissue capable of withstanding mechanical loadings. The duration of this bone apposition process can vary according to implant surface type, being around 4 weeks on blasting- and acid etching-obtained rough surfaces (24).

-Remodeling

Once formed, peri-implantary bone undergoes a remodeling process in which parallel fiber bone is mainly substituted by lamellar bone and bone architecture progressively adapts itself to its functional load (25). In this stage osteoblasts and osteoclasts work synergic ally, apposing and reabsorbing bone according to functional needs.

The bone-implant interface is under continuous remodeling and close contact between peri-implantary bone and the implant is essential to keep it functioning in the long-term.

## Osseointegration on bioinert and osseoconductive surfaces

Recent years have witnessed a progressive development of dental implants and much resources have been invested to improve implant surfaces. The bolt-shaped implant developed by Adell *et al*. was a pioneer in implantology and its use has proven good long-term clinical results (26). This titanium implant is characterized by its smooth or minim ally-rough (Sa < 0.5 μm) surface, resulting from drilling, which provides it with characteristic unevenness which are repeated showing a clear orientation across the implant (anisotropic surface).

This type of surface has been improved throughout the years with the creation of greater roughness so as to facilitate cell adhesion and thus accelerate implant osseointegration. While the first rough surfaces were obtained through additive particle processes such as those obtained by titanium plasma spraying, the most modern rough surfaces are obtained by subtraction methods. Among those most widely-used to obtain rough surfaces, aluminum oxide blasting, acid etching, surface oxidation and combinations of the aforementioned methods stand out (9).

These different procedures can produce mainly three types of implant surfaces: micro-structured rough surfaces (Sa = 0.5-1 μm), moderately rough surfaces (Sa = 1-2 μm), and highly rough surfaces (Sa > 2 μm) (27).

Results in literature confirm the greater effectiveness of rough surfaces relative to mechanized titanium ones, since a greater ratio of bone surface enters in contact with the implant (5), and they lead to improved (28) and quicker (24) osseointegration.

These results may be explained by the apparent different cell response in the earliest osseointegration stages. Firstly, surface roughness leads to significantly increased wetting and protein absorption, which in turn favor cell migration and adhesion (11).

However, Davies *et al*. hold that more favorable osseointegration is due to the clot’s fibrin scaffold’s greater adhesion force on rough vs. smooth surfaces (20). The fibrin scaffold allows osteoblast migration toward implant surface before these cells start to produce calcium phosphate crystals (hydroxyapatite). If fibrin’s adhesion capacity to implant surface exceeds the threshold, it shall be enough to allow osteoblasts to migrate through the scaffold and get in contact with implant surface. However, in mechanized titanium surfaces, no sufficiently stable bond occurs between it and fibrin so as to withstand the ‘weight’ of osteoblasts during their migration, thus producing separation between the implant and the fibrin scaffold. In this situation osteoblasts do not reach implant surface and new nuclei of bone formation will be placed closer to implant bed and far from implant surface. On the contrary, the fibrin scaffold on rough surfaces does not set free from the implant during osteoblast migration due to its tighter surface bond, thus allowing osteoblasts to reach the surface and start the bone apposition process.

Thus, difference can be made between mechanized titanium bioinert surfaces in which ‘contact osseointegration’ occurs (21) -i.e., progressive bone apposition from bed periphery to implant surface; and, on the other hand, osseoconductive surfaces, where the ‘bone neoformation’ can be observed -i.e., bone apposition contemporarily from implant surface and bed (22).

-A new paradigm -the biometric surface

Rough surfaces obtained through subtraction methods such as aluminum (Al2O3) particle blasting and acid etching prove improvements in in-vivo response relative to smooth surfaces (28). This procedure gets a surface topography characterized by concavities that form peaks and valleys that favor increased osteoconduction and, consequently, quicker bone growth with increased bone adhesion force (24).

However, the use of these surfaces still leads to reduced implant stability for the time period between the second and fourth week after placement. This is due to resorption of the bone initially in contact with the implant and, and also, to still slow bone neoformation, which fails to confer stiffness to the bone-implant bond. Consequently, increased amount of implant micro-movements may occur. Increased implant movements have been proven to determine the formation of a fibrous connective tissue and finally lead to its failure (29). This phenomenon has been observed to occur more frequently when the implant is subjected to functional load in its cicatrization stage, such as immediate loading. In these procedures the surface’s biological behavior gathers still more relevance, since the objective of obtaining an ideal surface also includes increasing implant stability during the critical stage of osseointegration.

Once the implant gets in contact with the bed after placement, osteoblasts from mesenchymal cells in the bone marrow form the first layers of calcium phosphate on the implant surface. This process, which is responsible for the formation of the first reticular bone, takes place by the process of bone resorption of the bed walls launched by osteoclastic cells.

A surface that provides quicker bone apposition in the first weeks after placement would allow lower reduction of implant stability during this critical stage and, thus, lower risk of osseointegration failure in an implant subjected to chewing load.

The use of coatings with similar composition to that of the bone are an attractive strategy to accelerate osseointegration during the earliest cicatrization stages. Particularly, calcium phosphate apatite has the same chemical composition as the mineral bone phase, so that complete acceptance by the organism and no inflammatory reaction occurs (30). Many researches have applied coatings on titanium implants by different techniques such as hydroxyapatite plasma spraying (31).

Although literature reports good clinical results for hydroxyapatite-coated implants and results are comparable to those achieved with titanium-surface ones (32), coatings obtained with certain techniques seem to have important drawbacks such as scarce adherence between implant titanium and the hydroxyapatite layer. In fact, additive techniques such as hydroxyapatite plasma spraying doesn’t allow formation of crystalline apatite but amorphous calcium phosphate due to high elaboration temperatures (33). The properties of this layer are not appropriate, since it is extremely soluble and titanium only achieves mechanical retention, no true adhesion.

Indeed, plasma spraying-obtained hydroxyapatite surfaces have proven scarce long-term clinical behavior, where -in spite of obtaining quick initial implant osseointegration- detachment of the osteophilic surface layer with time produces bacterial filtration into the interface and progressive osseointegration loss due to peri-implantitis (34).

New studies have recently proven that other methods to obtain phosphate calcium coating with higher homogeneity and chemical stability are possible (35). These new methods propose in-vitro apatite growth directly bound to the surface, thus achieving greater adherence and layer-thickness control. This can be achieved through surface, thermal and chemical treatments.

Pattanayak *et al*. completed apatite deposits based on the formation of a thick and amorphous gel of surface sodium titanate that, once immersed in ion-supersaturated serum (mainly calcium and phosphorus), can spontaneously generate a thin apatite layer that increases direct and structural connection with the structured bone (10). There are huge differences between this thermochemical treatment and those producing calcium phosphate deposits by plasma, since plasma starts from very high temperatures (6000-9000 ºC), under which the projected calcium phosphate is in plasma state and solidifies when launched to the dental implant. The first fact is that plasma solidification provides no crystalline calcium phosphate structure but an unstructured material known as calcium amorphous state, which cannot be known as apatite because it has no crystalline structure and is more similar to a frozen liquid. This is a very important aspect because in plasma-coated surfaces amorphous calcium phosphate dissolves much quicker than the crystalline phosphate. On the non-crystalline calcium phosphate layer also starts osteoblasts’ bone apposition process, although this neoformed bone will not get in direct contact with the implant surface when the calcium phosphate layer has dissolved; consequently, this phenomenon delays initial stages of the osseointegration process. Besides, calcium phosphate cooled down from such high temperatures is very fragile, since ceramic materials cannot withstand volume changes caused by such sudden temperature changes. Finally, the main limitation of plasma-formed layers is that they present no titanium-layer chemical bond, and their stability is mainly due to some mechanical clamp between titanium roughness and the amorphous mass of calcium phosphate. The biological consequence of this phenomenon is a bacterial microfiltration in the interface that in turn leads to osseointegration loss due to progressive peri-implantitis (34).

Obtaining calcium phosphate on implant surface by means of thermochemical treatments involves numerous advantages. Firstly, calcium phosphate is not organized amorphously but in a crystalline way, since it is formed by precipitation. This makes its structure (measured by X ray diffractograms) be the same as the calcium phosphate that forms bone mineral content (hydroxyapatite) (36), which provides a material with lower dissolving capacity in biological fluids and allows titanium chemical covalent bonds (37). This chemical bond renders excellent long-term stability and prevents all bacterial colonization between the calcium phosphate and the titanium (38).

Another important advantage of the thermochemical treatment relative to other hydroxyapatite obtaining methods is the obtained layer’s high mechanical resistance, since high temperature changes in plasma treatment are avoided (39).

**Figure 1:**
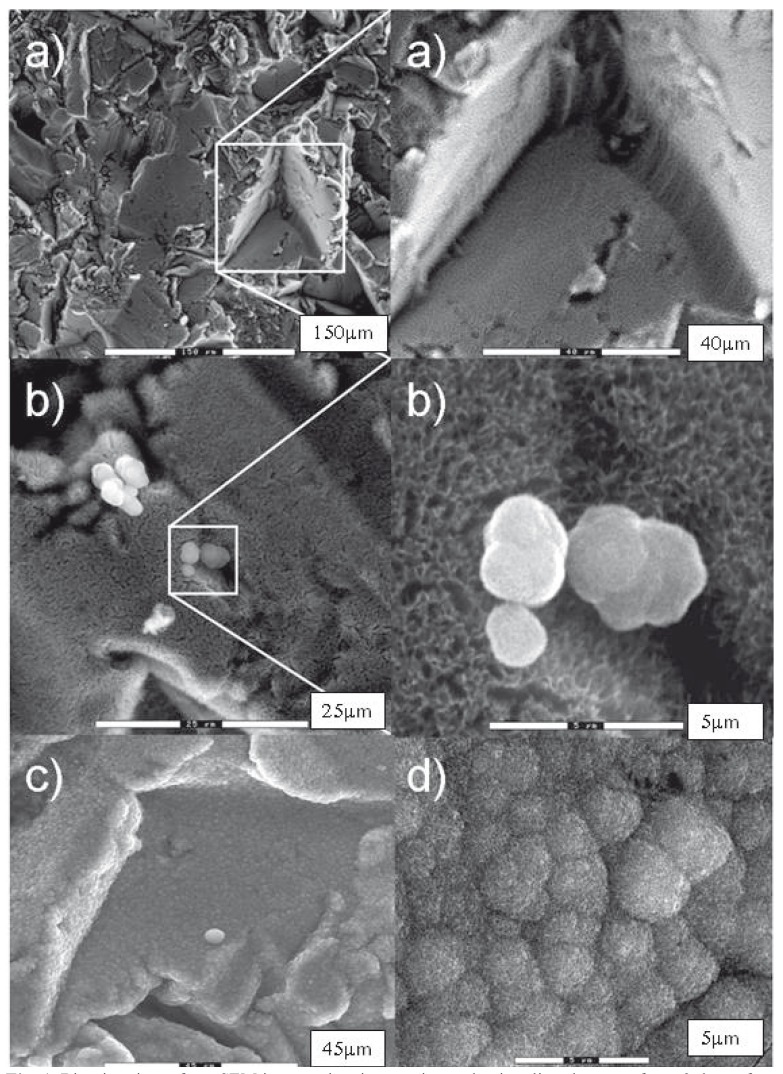
Biomimetic surface. SEM images showing apatite nucleation directly on surfaces 3 days after immersion.

This method can be said to provide a biomimetic surface, since the implant-covering sodium titanate layer can -thanks to Na+ ion bioactivity, and once it gets in contact with biological fluids- form on its own a hydroxyapatite layer without the need of osteoblasts taking part. This phenomenon has been proven both in-vitro and in-vivo by our research group, and accelerated osseointegration has been observed relative to untreated surfaces (28,37) (see Fig. 1).

Gil *et al*. have proven in histological studies in minipigs that thermochemical treatment of dental implant type-3 titanium surfaces can render full implant osseointegration within 4 weeks (37).

In their most recent study Gil *et al*. focused on the osseointegration capacity of 320 implants in minipigs, comparing bone response to different types of surface (37). The assayed surfaces were biomimetic surfaces obtained by combined aluminum oxide blasting and acid etching plus thermochemical treatment, rough surface obtained by aluminum oxide blasting, rough surface obtained by acid etching, and smooth surface as control. The implants used in this study were characterized by their 1.5-mm polished neck, 12-mm length and 1-mm thread pitch. Implant surface roughness was characterized first through electron microscopy, measuring surfaces contact angles and then the in-vivo test was completed by placing implants into mini pigs to which teeth were extracted 4 months before. Four implants of each type were placed in each animal, which were slaughtered 3 days, and 1, 2, 3 and 10 weeks after intervention to complete histological studies. Regarding surface characterization, no significant differences were observed in roughness values (Sa and Sm) between the biomimetic surface and the blasting-obtained rough surface. However, significant differences were found between these two and the acid etching-obtained rough surface (see  Table 2). The biomimetic surface proves lower contact angle relative to the blasting-obtained rough one, which shows up greater wetting and better behavior under blood contact.

**Table 2:**
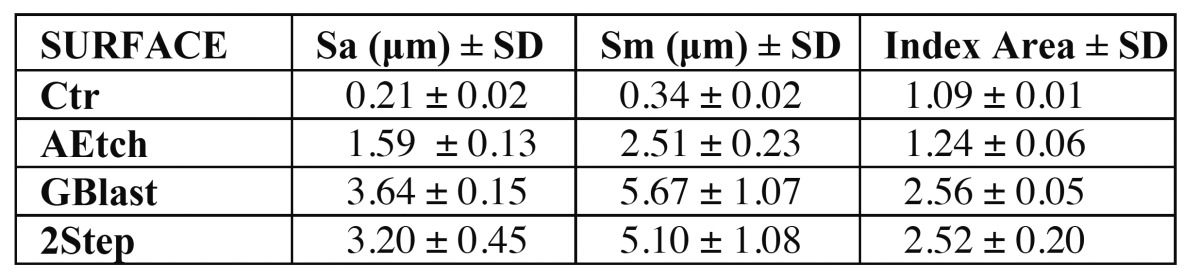
Values of surface roughness in mechanized (Ctr), acid etched (AEtch), aluminum oxide blasted (GBLast) and biomimetic surfaces obtained by blasting, acid etching and surface thermochemical treatment.

**Figure 2:**
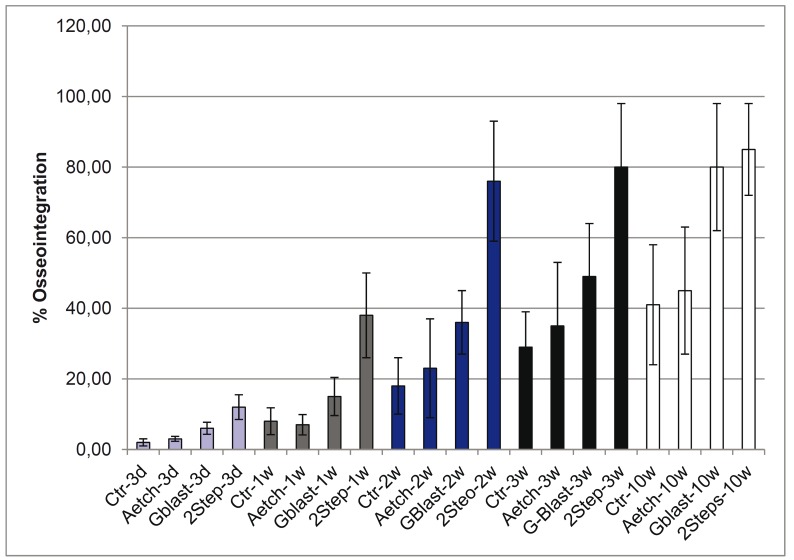
Ratio of bone in contact with the implant in the four types of surface researched 3 days, 1, 2, 3 and 10 weeks after placement. Crt: mechanized surface; AEtch: acid etching-obtained rough surface; Gblast: aluminum oxide blasting-obtained rough surface; 2 Step: rough surface obtained by aluminum oxide particle-blasting, acid etching and thermochemical treatment. Bone neoformation occurs significantly more quickly on the rough surface obtained by aluminum oxide particle-blasting, acid etching and thermochemical treatment, whose bone-implant contact (BIC) is over 70% and maximum at two and three weeks after stabilization, respectively.

Regarding bone-implant contact (i.e., the ratio of bone in contact with the implant), the biomimetic surface proves significantly higher values relative to the other surfaces 3 days, and 1, 2, 3 and 10 weeks after placement, though similar values are observed in blasting-obtained rough surface after 10 weeks (see Fig. 2).

**Figure 3:**
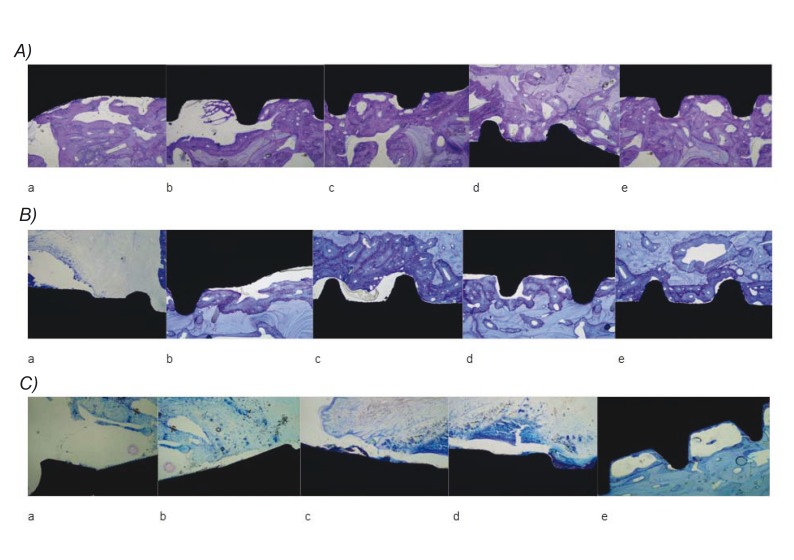
A) Acid-etching rough surface implant histology 3 day s(a), 1 (b), 2 (c), 3 (d) and 10 weeks (e) after placement. B) Histology of an rough surface implant obtained by aluminum oxide particle-blasting and acid etching 3 days, 1, 2, 3 and 10 weeks after placement. Surface shows accelerated ossification relative to the treatment including only acid etching. C) Histology of a rough surface implant obtained by aluminum oxide particle-blasting, acid etching and thermochemical treatment 3 days, 1, 2, 3 and 10 weeks after placement. Surface shows accelerated ossification relative to the treatment including aluminum oxide blasting and acid etching. Note the abundant presence of neoformed mature bone in contact with the implant surface.

This surface has presented surprisingly high osseointegration values in early cicatrization stages, being around 75% and 80% 2 and 3 weeks, respectively, after placement in this animal model. The biomimetic surface was the only one that clearly showed extensive areas of bone neoformation in direct contact with the implant after only one week of cicatrization (see Fig. 3). This phenomenon can be explained by the combination of osseoconductive phenomena provided by thermochemical treatment, which in turn naturally leads to the formation of calcium phosphate crystals on the implant surface once it gets in contact with biological fluids.

These encouraging results in this new surface can contribute to great clinical benefits for the application of immediate or early implant-loading protocols, however still need to be confirmed by clinical tests on humans, which are currently in developmental stages.

## Conclusions

Dental implant osseointegration is a phenomenon that has been studied for a long time. However, recent bioengineering has enabled us to understand the different biological events that characterize it -namely, protein adsorption, clot formation, granulation tissue formation, provisional matrix formation, interface formation, bone apposition and remodeling.

Protein adhesion has proven to play a key role in the earliest stages of osseointegration, where the presence of fibronectin and vitronectin favor osteoblastic cell line proliferation, while proteins such as TGF-α inhibit it.

Rough implant surfaces (Sa) over 1-2 μm lead to quicker osseointegration relative to micro-rough surfaces (Sa = 0.5-1 μm) due to the phenomenon of bone neoformation, where bone starts to form from implant surface toward the periphery at greater speed.

Implants presenting hydroxyapatite in their surface lead to accelerated osseointegration due to osteoblasts’ affinity to calcium phosphate. However, the surfaces produced up to date have presented long-term problems due to the bonding of this layer to the underlying titanium.

A biomimetic surface has been developed by means of thermochemical processing of titanium that allows the formation of a calcium phosphate layer in crystalline shape (hydroxyapatite), when the implant gets in contact with biological fluids. Studies in animals prove that this new surface can produce osseointegration in significantly shorter times relative to rough surfaces obtained by aluminum oxide blasting and acid etching. In-vivo studies show full implant osseointegration within 3 weeks, which would facilitate the use of immediate and early loading protocols.

These encouraging results need to be confirmed by clinical studies.
